# Costs and utilization of treatment in patients with hemophilia

**DOI:** 10.1186/s12913-015-1134-3

**Published:** 2015-10-26

**Authors:** Patrícia Rocha, Manuela Carvalho, Manuela Lopes, Fernando Araújo

**Affiliations:** Women’s Clinic, Centro Hospitalar São João, Alameda Prof. Hernâni Monteiro, 4200 Porto, Portugal; Department of Transfusion Medicine and Blood Bank, Center of Hemophilia, Centro Hospitalar São João, Porto, Portugal; Faculty of Medicine, University of Porto, Porto, Portugal

**Keywords:** Clotting factor concentrate, Cost of care, Disease management, Hemophilia, Hemophilia Centers, Portugal

## Abstract

**Background:**

Although hemophilia has a potentially high economic impact, there are no published estimates of healthcare costs for this disease in Portugal. The aim of this study was to evaluate costs of treatment and hospital utilization among patients with hemophilia A and B, with and without inhibitors, over a 3-year period in a Portuguese Comprehensive Care Hemophilia Centre. This is the first study on the financial impact of healthcare costs in patients with hemophilia in Portugal.

**Methods:**

This retrospective, observational study identified patients diagnosed with hemophilia A and B using medical and pharmacy electronic medical records and data from Centro Hospitalar São João, between January 2011 and December 2013. Patients with inhibitors were all high responders (>5 Bethesda Units [BU]). Severity was classified as mild, moderate or severe based on clotting factor levels. Two main outcomes were measured: (1) cost associated with hospital pharmacy claims (clotting factor) and (2) number of hospital visits/hospitalization.

**Results:**

A cohort of 103 patients were identified: 72 (69.9 %) with hemophilia A and 31 (30.1 %) with hemophilia B. Among these, five individuals were classified as patients with inhibitors (four with hemophilia A and one with hemophilia B). From the cohort of hemophilia A patients, 36 individuals (35.0 %) were identified as having severe disease; 20 (19.4 %) moderate; and 16 (15.5 %) mild. In the cohort of hemophilia B patients, 14 (13.6 %) were identified as having severe disease; 14 (13.6 %) moderate; and three (2.9 %) mild. The total mean aggregate cost per year (including clotting factor and hospital utilization) for patients with severe hemophilia B was €112,469, compared with €793 for mild hemophilia A. Clotting factor concentrate amounted for 90 % of total cost in severe cases and hospital utilization was also higher in these cases.

**Conclusions:**

Hemophilia treatment is expensive, particularly for patients with severe disease and especially if they develop inhibitors to replacement clotting factors. In our study, severe hemophilia is associated with greater annual total costs in both types of hemophilia (A = €77,587 and B = € 112,469). Patients with inhibitors have costs 3.3 times higher than patients without inhibitors. Age was not associated with significantly greater total costs (clotting factor and hospital visits/hospitalizations).

## Abstract (Portuguese)

**Introdução:**

Apesar da hemofilia possuir um impacto económico importante, não existem estimativas publicadas sobre os custos desta patologia em Portugal. O objectivo deste trabalho foi a avaliação da despesa em cuidados de saúde dos doentes com hemofilia A e B, no contexto de um Centro Compreensivo de Hemofilia Português. Este é o primeiro estudo do impacto financeiro dos custos do tratamento de doentes com hemofilia em Portugal.

**Métodos:**

Foram utilizados neste estudo retrospectivo e observacional os registos médicos electrónicos do Centro Compreensivo de Hemofilia do Centro Hospitalar São João, no período de Janeiro 2011 a Dezembro 2013. Foram identificados os doentes com hemofilia A e B assim como os doentes com inibidores. Os doentes com inibidores eram todos altos respondedores (>5 Unidades Bethesda). A hemofilia foi classificada como grave, leve ou moderada, consoante o nível de factor. Foram usados dois pontos (variáveis?): o custo associado ao concentrado de factor e o número de consultas/internamentos.

**Resultados:**

Foram identificados 103 doentes: 72 (69.9 %) com hemofilia A e 31 (30.1 %) com hemofilia B. Cinco doentes tinham inibidores (4 com hemofilia A e 1 com hemofilia B). No grupo de doentes com hemofilia A, 36 (35.0 %) são graves, 20 (19.4 %) moderados e 16 (15.5 %) leves. No caso da hemofilia B, 14 (13.65 %) são graves, 14 (13.6 %) moderados e 3 (2.9 %) leves. A média anual de custos (incluindo os concentrados de factor da coagulação e os episódios registados no hospital) variou entre €112,469 para a hemofilia B grave, e €793 para a hemofilia A leve. Nos doentes com hemofilia grave, os concentrados de factores da coagulação foram responsáveis por 90 % dos custos e as consultas externas, episódios de urgência e internamentos foram também mais elevados.

**Conclusões:**

O tratamento da hemofilia possui custos elevados, nomeadamente nos doentes com hemofilia grave e/ou com inibidores. No nosso estudo, os doentes hemofílicos com maior gravidade estão associados com custos totais anuais mais elevados nos dois tipos de hemofilia (A = €77,587 e B = € 112,469) e os doentes com inibidores têm custos 3.3 vezes mais elevados do que os doentes sem inibidores. Além disso, não se identificou uma associação estatisticamente significativa entre a idade e os custos totais (concentrado de factor e utilização hospitalar).

## Background

Hemophilia is a rare genetic disease linked to the X-chromosome. The presence of specific genetic mutations results in an inability to produce the clotting factors needed to stop bleeding. The two most common types are hemophilia A, a deficiency of clotting Factor VIII; and hemophilia B, a deficiency of clotting Factor IX. Hemophilia A affects one in 5000–10,000 and hemophilia B one in 25,000–30,000 male births [1]. Although hemophilia is largely an inherited bleeding disorder, approximately one-third of patients present with no prior family history; in these patients, hemophilia results from a *de novo* gene mutation [2].

The treatment of hemophilia is based on clotting factor replacement therapy. The most commonly used treatments for hemophilia are “on-demand” therapy and factor prophylaxis. During a bleeding episode, clotting factor can be given “on-demand” to control and stop the bleeding; however, this type of therapy does not prevent the development of hemarthrosis (a bleeding into joint spaces) or its consequences. During prophylactic treatment, patients receive regular factor concentrate replacement therapy, which can lead to better clinical outcomes, with a lower overall impact on quality of life; however, this type of treatment is more expensive than “on-demand” therapy [3, 4].

Over the past 30 years, the life expectancy of people with hemophilia has significantly increased, largely due to advances in medical care; the introduction and accessibility of safe and effective factor replacement therapies; and improvements in treatment of infectious diseases. For example, in Sweden between 1969 and 1980, life expectancy for a patient with hemophilia receiving factor replacement therapy was only 58 years [5]. Today, with the new model of treatment care, life expectancy is similar to that of people without hemophilia, which creates new, complex clinical issues not all directly related to hemophilia [6].

These advances have been achieved at high financial costs, and hemophilia remains one of the most expensive and challenging diseases to manage. Clotting factor costs amount to 50–90 % of the total healthcare costs for hemophilia treatment, depending on severity and type [7, 8]. In the United States, Globe et al. [9] estimated that the annual medical cost for a patient’s hemophilia care was $132,102, with clotting factor use accounting for an average of 72 % of total costs. Excellent clinical results have been demonstrated with high-dose prophylaxis factor therapy; however, because of the high costs associated with this treatment, its use should be assessed based on a cost-benefit evaluation [10].

Furthermore, some patients with hemophilia develop inhibitors to replacement clotting factors, which renders treatment ineffective and results in the requirement for a different treatment strategy [11]. It is estimated that inhibitors develop in 25–30 % of severe hemophilia A patients and 3–5 % of severe hemophilia B patients [12]. In the United States, Guh et al. [13] found that the total annual cost of treatment for a hemophilia A patient developing inhibitors was 4.8 times higher than for a patient without inhibitors [14, 15]. In these cases, immune tolerance induction is an option; it involves the use of very high doses of clotting factor, with the aim of desensitizing the patient to the novel protein. This treatment strategy can eradicate persistent inhibitors in some patients with severe hemophilia A [16, 17]. However, the requirement for bypassing agents and immune tolerance induction in hemophilia patients who develop inhibitors further increases overall treatment costs [18].

No previous studies on hemophilia costs have been conducted in Portugal. The Portuguese Association of Hemophilia (APH) [19] has reported between 700 and 800 registered members; however, this may be an underestimation and the overall prevalence of hemophilia in Portugal is unknown, as there is no national registry. According to the final report of the national working group established by the government “Despacho N° 4319/2013, de 15 de Março de 2013”[20], approximately 2400 people in Portugal are thought to have inherited coagulopathies. The difference between these figures may be due to the fact that people with mild or moderate hemophilia rarely experience spontaneous bleeding and may therefore not be identified unless they have an acute event, such as surgery or an accident. Alternatively, some individuals do not want to be registered with a patient association.

Recently, the Direcção-Geral de Saúde (DGS) [21], under the National Program for Rare Diseases, commenced data collection for a nationwide registry for these rare diseases via the distribution of the “Rare Disease Patient Card”; therefore, it is expected that in the near future, an effective and accurate national database will be accessible.

Data from the Portuguese National Authority to Drugs and Devices (INFARMED) [22] show an increase of 167.5 % in hospital costs for hemostatic drugs between 2008 and 2013, which represents approximately 4 % of total national costs of hospital drugs (Table 1). Based on the knowledge that there are approximately 800 people in Portugal with hemophilia, it can be calculated that an average of €44,134 is spent by each patient on clotting factors per year. According to the Organization to Economic Co-operation and Development (OECD)[23], expenses on health per capita (private and public) was €1924 in Portugal, representing about 10.2 % of Gross Domestic Product (GDP).

**Table 1:** Total hospital drug costs in Portugal

	2008	2009	2010	2011	2012	2013
Total drug costs	573,228	670,028	993,787	1,012,518	1,017,942	974,824
Hemostatic drugs	13,200	21,622	36,793	37,370	36,861	35,307
% of total national costs	2.3 %	3.2 %	3.7 %	3.7 %	3.6 %	3.6 %

Source: INFARMED Unit: M€

The purpose of this study was to evaluate the characteristics and health system costs in patients with hemophilia by type, age and severity, with and without inhibitors, over a three-year period, at the Hemophilia Comprehensive Care Center (CCC) of the University Hospital, Centro Hospitalar São João - Entidade Pública Empresarial (CHSJ –EPE).

## Methods

A retrospective, observational study was performed, using medical and pharmacy electronic medical records and data from CHSJ-EPE, between January 2011 and December 2013. Patients for this study were selected from our Hemophilia CCC database if they had at least one episode registered in our hospital in that period; Patients were identified according to their hemophilia type, severity, presence/absence of inhibitors, and age.

Patients with inhibitors were all high responders (>5 BU). Hemophilia severity was classified as mild (5–40 % of normal factor level), moderate (1–5 % of normal factor level), or severe (<1 % of normal factor level) [24]. There were six groups established according to type and severity of hemophilia: (i) patients with severe hemophilia A; (ii) patients with moderate hemophilia A; (iii) patients with mild hemophilia A; (iv) patients with severe hemophilia B; (v) patients with moderate hemophilia B; (vi) patients with mild hemophilia B. Patients were also divided by type (A or B) and age: Pediatrics (<18 years) and adults. Additionally, patients who were human immunodeficiency virus (HIV) and/or hepatitis C virus (HCV)-positive were identified.

Two main outcomes were measured from January 2011 through December 2013: (1) cost associated with all hospital pharmacy claims (clotting factor) and (2) number of hospital visits/hospitalization.

Using the CHSJ-EPE Business Intelligence system, patient data related to drug costs and hospitalization/hospital visits were collected. Because in Portugal hospital visits, inpatient admissions and emergencies are each associated with a specific national funding price, the costs resulting from hospital visits were calculated based on hospital reimbursement. Transportation and diagnosis costs were considered minimal and were therefore not included in the analysis.

Costs were annualized by computing the sum of total costs for each patient and dividing by 3. Total costs were defined as the sum of the two aforementioned costs.

Statistical analysis was performed using SPSS-22 (Statistical Package for the Social Sciences). Differences in means were tested using the Mann-Whitney U test. The Kruskal-Wallis test was used to compare more than two groups. These non-parametric tests were used because data were not normally distributed.

## Results

A total of 103 patients were identified; 72 (69.9 %) had hemophilia A and 31 (30.1 %) had hemophilia B (Table 2). Overall, five (4.9 %) expressed inhibitors (four patients with hemophilia A and one patient with hemophilia B). In the hemophilia A cohort, 36 patients (35.0 %) had severe disease; 20 (19.4 %) moderate disease; and 16 (15.5 %) mild disease. In the hemophilia B cohort, 14 patients (13.6 %) had severe disease; 14 (13.6 %) moderate disease; and three (2.9 %) mild disease. A small number of patients (*n* = 8; 7.8 %) were HIV-positive and 22 patients HCV-positive (21.4 %). All patients who were cases HIV-positive or HCV-positive were adults (>38 years for HIV and >26 years for HCV).

**Table 2:** Demographic characteristics

Characteristic	Number (%) of patients (*N* = 103)
Hemophilia type	
A	72 (69.9)
B	31 (30.1)
Hemophilia Severity	
Severe	50 (48.5)
Moderate	19 (18.5)
Mild	34 (33.0)
Patients with inhibitors	
Yes	5 (4.9)
No	98 (95.1)
Patients with infections	
HIV	8 (7.8)
HCV	22 (21.4)
Age, (median, years)	
Pediatric	27 (10)
Adult	76 (41)

On average, each patient had 13 consultations and six days of hospital admissions per year. During the study period, a 19.8 % reduction in consultations and 39.8 % in day hospital admissions was seen. This was partly due to the adoption of a prophylactic approach including all children with severe hemophilia, as well as improvements in home therapy following participation in a patient and family training program for self-administration of clotting factors, which has advantages for both patients and the healthcare system. Of the 21 surgical admissions recorded, most were in adult patients (*n* = 19). Surgeries related to “diseases and disorders of musculoskeletal system and connective tissue” (namely, orthopedic surgery of the hip or knee, performed in adults) were 33.3 %, followed by “diseases and disorders of the nervous system” (23.8 %), and “diseases and disorders of ear, nose, mouth and throat” (19.0 %), which were more frequent in children and teenagers than adults.

The overall healthcare annual costs are shown in Table 3. Costs for patients in the severe group were considerably higher than costs for those in the mild/moderate groups. For patients identified with hemophilia A, the annual mean healthcare cost was €77,587 for severe patients, €8495 for moderate and €793 for those with mild hemophilia (*P* = 0.000). Individuals with severe hemophilia B had the highest total healthcare costs (mean = €112,469). The unexpectedly high value obtained for patients with mild hemophilia B (€10,217 compared with €793 for patients with mild hemophilia A) is likely related to the very small number of patients in this group (*n* = 3). Only one of these patients had previously suffered from hemorrhagic transformation of cerebral septic emboli and, as a result of infectious endocarditis, had undergone surgery to insert a prosthesis mechanical valve in the heart. This procedure, and the hospitalization-associated costs, significantly increased the overall clotting factor costs. In both severe hemophilia A and severe hemophilia B patients, clotting factor accounted for more than 90 % of the total treatment cost (92 and 93 %, respectively).

**Table 3:** Annualized costs, per year, by type and severity (€; Euros)

	Hospital utilization	Clotting factor	Total costs	% Clotting factor	*P*-value
Type A					
Severe	6486	71,101	77,587	92 %	0.000
Moderate	2469	6026	8495	71 %	
Mild	0506	0287	0793	36 %	
Type B					
Severe	7846	104,623	112,469	93 %	0.000
Moderate	0775	2108	2883	73 %	
Mild	1913	8304	10,217	81 %	

*P* values were computed using the KrusKal Wallis test

As expected, statistically significant differences were found between patients with and without inhibitors in total annual costs (Table 4). Costs were higher for patients with inhibitors (€134,032) than for patients without inhibitors (€40,138, *P* = 0.03). Although, the only patient with severe hemophilia B and inhibitors was a child, the average annual clotting factor cost for this patient was seven times higher than for patients with hemophilia B without inhibitors. This patient had an anaphylactic reaction to Factor IX concentrate treatment and an intracranial hemorrhage, which raised the costs in this group. Accordingly, due to the risk of immune tolerance and the previously documented low success rate of Factor IX, it was decided to maintain prophylaxis with recombinant Factor VIIa. During this period studied, there was only one patient (a child with severe hemophilia A) documented to have immune tolerance therapy, which was associated with a total annual cost of €317,415.

**Table 4:** Annualized total costs by inhibitor status (€; Euros)

	Total cost	*P*-value
With inhibitor (*n* = 5)	134,032	0.030
Without inhibitor (*n* = 98)	40,318	

*P* value was computed using the Mann-Whitney U test

A key finding in this analysis is that no statistically significant differences were found between pediatric and adult patients in both types of hemophilia, despite therapy being related to the patient’s weight (Table 5). For pediatric patients with hemophilia A, annual costs were €37,805 while for adults were €49,338 (*P* = 0.471). For patients with hemophilia B, these costs were €104,618 and €43,173 (*P* = 0.815), respectively.

**Table 5:** Annualized total costs, by type and age (€; Euros)

	Total cost	*P*-value
Type A		
Adult (*n* = 50)	49,338	0.471
Pediatric (*n* = 22)	37,805	
Type B		
Adult (*n* = 26)	43,173	0.815
Pediatric (*n* = 5)	104,618	

*P* value was computed using the Mann-Whitney U test

## Discussion

The results of our study confirm those of previous studies on hemophilia costs previously conducted in Europe; in Italy, Kodra et al. [25] estimated mean direct healthcare costs per patient with hemophilia to be €109,768; and in Belgium, Henrad et al. evaluated this cost to be €97,336 [26].

Our findings reveal that a small proportion of patients with hemophilia (*n* = 5; 4.9 %) developed inhibitors to clotting factor products and required treatment with bypassing agents. However, given the associated costs for these additional treatments, these patients significantly contribute to overall global expenditure. In our study, patients with inhibitors accounted for 3.3 times the annual cost of patients without inhibitors. It should be noted that there are now more children with hemophilia who are reaching adulthood, at which stage they begin individualized prophylaxis treatment. As the cohort of young patients on prophylaxis treatment grows older and larger, the demand for factor concentrates will likely increase, which could lead to increased factor costs as a result.

The total annual costs were not statistically different between adults and children in both types of hemophilia. There could be three possible explanations for the similar costs: as the vast majority of children in this study were teenagers (median age of ten years), their body weight would be comparable with adults; the different pharmacokinetics of clotting factors in children, which implies a higher consumption clotting factor per kg; and the model used, as prophylaxis is used in 100 % of children and teenagers versus 40–70 % in adults with severe hemophilia.

Because the number of patients with HIV or HCV infection was small, we did not carry out an analysis comparing infected versus non-infected patients with hemophilia. Guh et al. [27] previously demonstrated that mean cost of hemophilia treatment is independent of blood-borne viral infection status. However, Tencer et al. [28] concluded that annual medical costs (clotting factor, prescription drugs, inpatient services and outpatient services) were 59 % greater in patients with hemophilia who also had HIV and/or HCV compared with those with hemophilia alone.

There are three important limitations to our study. The first limitation is that hemophilia is a rare disease; consequently, it is difficult to collect data on a large cohort. Because of this restriction, Farrugia et al. [29] underlined the role of observational analysis as a method to increase the evidence base of hemophilia treatment. The second limitation is that this study was conducted on a small scale, with data only being collected from one hospital (including the outpatient or pharmacy departments). In addition, it did not take into consideration other resources used in homecare to manage aspects of the condition such as breakthrough bleeding, which does not require a clinic visit (excluding clotting factors, which were integrated to the calculations). The final limitation is that the hospital prices used to calculate the total costs are not the real costs, but hospital reimbursement prices. However, in the absence of a reliable cost calculation, this was considered to be an appropriate alternative for an approach in which factor concentrates represent more than 90 % of overall expenses (in severe patients and patients with inhibitors).

In the United States, where health expenses per capita represent 17.7 % of GDP, hemophilia care is delivered in highly specialized centers, designated as Hemophilia CCCs, which treat approximately 70 % of people with this disorder [30]. Hemophilia CCCs offer comprehensive, multidisciplinary hemophilia services, treat a large number of patients, and have long-standing experience in the management of this disease, which is needed to prevent and manage complications, especially in severe patients [31]. Furthermore, the increasing prevalence of age-associated diseases, such as cardiovascular pathologies or osteopenia, will pose additional challenges in the treatment of these patients [32].

This is the first study on the financial impact of healthcare costs in patients with hemophilia in Portugal. Despite the rarity of this disease, the high costs associated with its treatment justify the concentration of resources and assurance of the most cost-effective care [33, 34], and therefore have been integrated into a national strategy for a “more coherent hospital network”.

The Public Health Ministry of Portugal has recently approved the guidelines for hemophilia treatment [35], which describe the clinical protocols and the range of quality indicators that should be evaluated periodically. CHSJ-EPE was recognized by the European Hemophilia Network (EUHANET) [36] as a European Hemophilia CCC, according to the performance and differentiation of services. This network has 84 centers in 26 countries. The future development of this project will require the expansion of measures based on economic, clinical and quality outcomes [37].

## Conclusions

Severe hemophilia is associated with greater annual total costs in both types of hemophilia (A = €77,587 and B = € 112,469). Patients with inhibitors accounted for 3.3 times the annual cost of patients without inhibitors. In addition, age is not associated with significantly bigger total costs (clotting factor and hospital visits/hospitalizations).

The high costs of hemophilia treatment, coupled with the requirement for highly specialized therapies, present a unique challenge for care management and reimbursement considerations for health planning.

## Abbreviations

APH: Portuguese Association of Hemophilia
BU: Bethesda units
CCC: Comprehensive Care Center
CHSJ-EPE: Centro Hospitalar São João, Empresa Pública Empresarial
DGS: Direcção-Geral da Saúde
EUHANET: European Hemophilia Network
GDP: Gross Domestic Product
HCV: Hepatitis C Virus
HIV: Human Immunodeficiency Virus
INFARMED: Portuguese National Authority to Drugs and Devices
IU: International Units
OECD: Organization to Economic Co-Operation and Development
SPSS: Statistical Package for the Social Sciences
